# OMRT-11. The effect of microenvironment on glioblastoma stem cells therapeutic resistance

**DOI:** 10.1093/noajnl/vdab070.035

**Published:** 2021-07-05

**Authors:** Tamara Lah Turnsek, Barbara Breznik, Bernarda Majc, Metka Novak, Andrej Porčnik, Anamarija Habič, Mateja Mlinar, Roman Bošnjak

**Affiliations:** 1 National Institute of Biology, Ljubljana, Slovenia; 2 The Jožef Stefan International Postgraduate School, Ljubljana, Slovenia; 3 Department of Neurosurgery, University Medical Centre, Ljubljana, Slovenia

## Abstract

Epithelial-to-mesenchymal transition (EMT) is an essential molecular and cellular process in physiologic processes and invasion of various types of carcinoma and glioblastoma (GBM) cells. EMT is activated and regulated by specific endogenous triggers in complex network of intercellular interactions and signaling pathways. The hallmark of cancer-linked EMT are intermediate states that show notable cell plasticity, characteristic of cancer stem cells (CSCs), including glioblastoma stem cells – GSCs. GSCs resistance to irradiation (IR) and temozolomide (TMZ) chemotherapy is responsible for early relapses, even at distant brain sites. As GSCs are mostly homing to their “niches” as slowly-dividing GSC-subtype, mimicking a proneural-like non- invasive phenotype PN-genotype, we assume that this, by undergoing an EMT-like transition, GSCs are-reprogrammed to an invasive mesenchymal (MES) GBs/GSCs phenotype in a processes, called PMT (1). However, it is not known, if and by which environmental cues within the niche, this transition of GSCs is induced in vivo. In this work, we are presenting the transriptome data obtained when we exposed GSC spheroids to irradiation alone, TMZ alone and to the combined treatment *in vitro* and compared their differential genetic fingerprints related to EMT/PMT transition to the GSCs PMT transition, when embedded in their natural microenvironment in the GBM organoid model. The differential gene expression upon GSCs therapeutic perturbation (when alone and *vs* in the tumoroid microenvironment) will reveal the effects of the major candidate genes, associated with micronevironmendt stromal cells and matrix are contributing their observed EMT/PMT transition of GSCs in vivo.

•1. Majc, B., Sever, T., Zarić, M, Breznik, B., Turk, B, Lah Turnšek, T. Epithelial- to-mesenchymal transition as the driver of changing carcinoma and glioblastoma microenvironment. DOI: 10.1016/j.bbamcr.2020.118782

